# A common mechanism explains the induction of aerobic fermentation and adaptive antioxidant response in *Phaffia rhodozyma*

**DOI:** 10.1186/s12934-018-0898-7

**Published:** 2018-04-03

**Authors:** Anahí Martínez-Cárdenas, Cipriano Chávez-Cabrera, Jazmín M. Vasquez-Bahena, Luis B. Flores-Cotera

**Affiliations:** 1 Department of Biotechnology and Bioengineering, Cinvestav-IPN, Av. Instituto Politécnico Nacional 2508, Col. San Pedro Zacatenco, 07360 Mexico City, Mexico; 2 Avi-mex Laboratory S.A de C.V, Trigo 169, Col. Granjas Esmeralda, 09810 Mexico City, Mexico; 3 Present Address: College of Science and Technology Studies of the State of Michoacán, Loma de las Liebres 180, Fraccionamiento Lomas del Sur, 58095 Morelia, Michoacán Mexico

**Keywords:** Redox signaling, Aerobic glycolysis, Crabtree effect, Warburg effect, Redox homeostasis

## Abstract

**Background:**

Growth conditions that bring about stress on *Phaffia rhodozyma* cells encourage the synthesis of astaxanthin, an antioxidant carotenoid, which protects cells against oxidative damage. Using *P. rhodozyma* cultures performed with and without copper limitation, we examined the kinetics of astaxanthin synthesis along with the expression of *asy*, the key astaxanthin synthesis gene, as well as *aox*, which encodes an alternative oxidase protein.

**Results:**

Copper deficiency had a detrimental effect on the rates of oxygen consumption and ethanol reassimilation at the diauxic shift. In contrast, copper deficiency prompted alcoholic fermentation under aerobic conditions and had a favorable effect on the astaxanthin content of cells, as well as on *aox* expression. Both cultures exhibited strong *aox* expression while consuming ethanol, but particularly when copper was absent.

**Conclusion:**

We show that the induction of either astaxanthin production, *aox* expression, or aerobic fermentation exemplifies the crucial role that redox imbalance plays in triggering any of these phenomena. Based on our own results and data from others, we propose a mechanism that rationalizes the central role played by changes of respiratory activity, which lead to redox imbalances, in triggering both the short-term antioxidant response as well as fermentation in yeasts and other cell types.

## Background

*Phaffia rhodozyma* (sexual state, *Xanthophyllomyces dendrorhous*) is a basidiomycetous yeast capable of synthesizing astaxanthin, a highly effective antioxidant carotenoid pigment [1–3]. Astaxanthin effectively protects *P. rhodozyma* cells against oxidative damage mediated by reactive oxygen species (ROS) such as superoxide ion (O_2_^·−^), singlet oxygen (^1^O_2_), and hydrogen peroxide (H_2_O_2_) irrespective of whether these are found in the yeast natural habitat or generated by the yeast itself via its intracellular oxidative metabolism [4–7]. *P. rhodozyma* possesses a broad range of genes related to antioxidant defense, a number of which are shared by all eukaryotes, but lacks or exhibits low activity of the cytosolic superoxide dismutase (SOD1 or Cu/Zn SOD). Nevertheless, astaxanthin is considered a primary, uncommon and critical antioxidant protection for this yeast [8, 9].

Moreover, several studies have shown that low nutrient availability in the culture medium (e.g., nitrogen, phosphate, or copper), among other environmental factors, can promote the synthesis of astaxanthin by *P. rhodozyma* [10–15]. In particular, Flores-Cotera and Sánchez [12], using a wild-type strain of *P. rhodozyma,* found that decreasing copper concentrations in the culture medium (from 3.2 to 0.0 μM) significantly increased the intracellular astaxanthin content from 220 to 287 μg g^−1^ whereas the biomass concentration decreased. *P. rhodozyma* cells accumulate astaxanthin whenever they are faced with oxidative stress; thus, at the lower copper levels, it can be assumed that yeast cells were confronted with further oxidative stress. Copper is a key trace element for the function of several enzymes involved in processes such as energy metabolism, mitochondrial respiration, and DNA synthesis. As such, it is an essential metal for cell growth and development in all living organisms [16–18]. Cytochrome c oxidase (CcO or Complex IV), the terminal oxidase of the mitochondrial respiratory chain, possesses two copper catalytic centers (Cu_A_ and Cu_B_) that are highly conserved in eukaryotic cells from yeast to higher organisms [19, 20]. In particular, the catalytic reduction of dioxygen to water by CcO depends on a cytochrome Cu_B_-heme a_3_ [21–23]. For these reasons, the analysis of events involved in triggering astaxanthin synthesis under copper deficiency may help to gain insight into the mechanism leading to oxidative stress in this yeast, as well as in other cell types under similar conditions.

*Phaffia rhodozyma*, as with other carotenogenic yeast species including *Cryptococcus*, *Rhodotorula*, and *Sporobolomyces*, as well as all plants, most fungi, algae, and some protists, possesses an alternative oxidase (AOX) which is insensitive to inhibitors of cytochromes such as cyanide and antimycin A [6, 24–26]. AOX catalyzes the intramitochondrial oxidation of ubiquinol (QH_2_) coupled with the reduction of dioxygen to water. This reaction does not generate proton-motive force but allows an ongoing transfer of electrons when the main respiratory chain is hampered [27]. Thus, AOX provides an alternative pathway for the passage of electrons and the continuity of growth under such conditions [28–31]. It has been shown that antimycin A, an inhibitor of electron transport from cytochrome b to c in the respiratory chain, is able to trigger astaxanthin synthesis in *P. rhodozyma* [32]. In the presence of antimycin A, the synthesis of astaxanthin occurred along with a shift from cyanide sensitive respiration (cytochrome) to cyanide insensitive respiration. Accordingly, it was proposed that AOX activation is closely associated with astaxanthin synthesis. Conversely, a subsequent study showed that *aox* deletion instead promotes astaxanthin production [33].

The previously mentioned examples with inhibitors and nutritional limitations show that *P. rhodozyma* accumulates astaxanthin intracellularly when faced with stressful conditions; therefore, this yeast represents an ideal model for in vivo studies concerning the events that lead to cellular oxidative stress, as intracellular astaxanthin accrual would serve as an exceptional marker of such stressful conditions. The first aim of this study was to examine the effect of copper deficiency on the antioxidant response of *P. rhodozyma*, as determined by the production of astaxanthin as well as the expression of the key carotenogenic gene, astaxanthin synthase (*asy*, also known as *crtS*), and *aox*. For this purpose, cultures of the yeast grown under copper deficiency (−Cu) were compared with those grown in the presence of copper (+Cu). The second aim was to propose a hypothetical mechanism that rationalizes the central role of redox imbalances to induce aerobic fermentation and the short-term antioxidant response of this yeast under changing environmental conditions. Aerobic fermentation, astaxanthin production, and *aox* expression were used to exemplify the crucial role that hindrances to oxygen consumption, and hence, redox imbalances, play in the activation of these processes. The possible relevance of the mechanism proposed to other cell types is briefly argued.

## Methods

### Strain and culture conditions

A wild-type strain, *P. rhodozyma* NRRL-Y-10922 (sexual state *X. dendrorhous*), was used throughout this work. The culture medium for preservation was YM-agar comprised of glucose (1.0%), yeast extract (0.3%), malt extract (0.3%), bactopeptone (0.5%), and agar (1.5%) (Becton, Dickinson & Co., Sparks, MD, USA).

The inoculum was generated in 250 ml Erlenmeyer flasks containing 32 ml liquid medium. The lyophilized yeast was suspended in YM broth (200 μl), spread on Petri plates containing YM agar, and incubated at 22 °C for 72 h. A yeast colony was picked, seeded on another Petri plate, and incubated for a further 72 h. Then, three Erlenmeyer flasks containing YM liquid medium (32 ml) were inoculated each with a loop of cells taken from the latter plate. The flasks were incubated in an Innova 43 orbital shaker (New Brunswick, NJ, USA) at 120 rpm, 22 °C, for 18–20 h.

All experiments were performed in an Applikon fermenter system (Delft, The Netherlands) fitted with a 2.5 l nominal vessel and 2.1 l operating volume. The culture conditions were as follows: aeration 1 vvm (standard conditions), 700 rpm, 22 °C, and the pH was left free until 4.5, at which time it was automatically controlled with a 5% NaOH solution. The total content of the three flasks was centrifuged and washed twice with 0.9% NaCl solution and used to inoculate the fermenter. Concentrations of 7 and 0.12 μM (this estimated from the copper content of water and the salts added) were used in the cultures with (culture +Cu) and without copper (culture −Cu), respectively. The medium components were as follows (g l^−1^); 30 sucrose, 1.6 (NH_4_) 2SO_4_, 0.39 Na_2_HPO_4_, 0.08 MgSO_4_·7H_2_O, 0.18 K_2_SO_4_, 0.02 CaCl_2_·2H_2_O, 0.002 FeSO_4_·7H_2_O, 0.012 ZnSO_4_·7H_2_O, 0.0006 MnSO_4_·H_2_O, 0.002 CoSO_4_·7H_2_O, 0.00013 H_3_BO_3_, 0.00053 Na_2_MoO_4_·2H_2_O, 1.45 NaCl, 0.35 Na_2_SO_4_, and 12 ml of vitamin solution. The vitamin solution contained (mg l^−1^): 150 calcium pantothenate, 76 thiamine, 4 biotin, 7.6 cyanocobalamin, 7.6 myo-inositol, 38 pyridoxine chloride, 76 riboflavin, and 450 nicotinamide (all vitamins from Sigma-Aldrich, St. Louis, MO, USA) dissolved in deionized water. For the fermenter experiments, deionized water with a resistivity of 18.2 MΩ cm (Milli Q Advantage 10, Merck KGaA, Hesse, Germany) was used. Preliminary experiments (n > 8) without copper supply indicated that the culture was sensitive to the presence of trace quantities of the metal such that a careful cleaning of all glassware and water was required for the −Cu experiments. Two replicas at each experimental condition were performed; the results are from a representative experiment to each culture condition.

### Biomass, sugars, and ethanol

The cell pellet obtained from 1–2 ml samples of fermented broth (depending on the culture age) was centrifuged at 1500*g* for 5 min, washed once with distilled water and then recovered in distilled water for biomass assessment. Biomass was determined by assessing dry cell weight as previously reported [11]. Ethanol was determined by chromatography using supernatants obtained by centrifugation of culture broth samples (0.5 ml). A Varian CP-3380 gas chromatograph (Palo Alto, CA, USA) with a flame ionization detector fitted with a ZB-FFAP column (Agilent, 15 m × 0.53 mm x 1 μm) was used. Operating conditions: 300 and 200 °C temperature at the flame ionization detector and injector, respectively, and 1 ml min^−1^ nitrogen (99.99%) flow rate. The standard curve was obtained with high performance liquid chromatography (HPLC) grade ethanol. Reducing sugars were determined by colorimetry using the DNS method [34]. The specific sugar uptake rate was calculated as the quotient between sugar uptake rate (g l^−1^ h^−1^) and biomass concentration (g l^−1^). The sugar uptake rate was determined assuming a continuous function of sugar concentration with time, and according The Mean Value Theorem.

### Extraction and determination of carotenoids

The quantification of pigments was performed according to a modified method previously described [35]. Cell pellets obtained by centrifugation (~ 2.5 mg dry cells) were lyophilized for 3 h, and the dried cells were then mixed with 0.5 ml glass beads (0.5 mm diameter) and pulverized with a pestle (15 s), to which 0.2 ml of 0.01 M NaH_2_PO_4_ and 1 ml of dimethylsulfoxide at 58 °C were added and incubated for 1 min at 58 °C. Next, each sample was mixed by vortexing for 45 s to aid cell wall breakdown and extracted with 5 ml hexane:ethyl acetate (1:1) mixture and vortexed for 1 min. The resultant emulsion was centrifuged at 1500*g* for 5 min. The absorbance of the organic phase containing the carotenoids was determined at 480 nm using a Biowave II spectrophotometer (Biochrom US, Holliston, MA, USA). Carotenoid concentration was determined using an extinction coefficient of 2100 and the equation:

$$\begin{aligned} {\frac{\mu g}{ml}} & = & {\frac{10000\,*\,A\,*\,Vs}{{Vm\,*\,E_{1 \text{cm}}^{1\% } }}} \hfill \\ \end{aligned}$$

where A is the absorbance at 480 nm; Vs is the recovered solvent volume (ml); Vm is the sample volume (ml); and E_1 cm_^1%^ is the specific extinction coefficient of astaxanthin (2100).

### Determination of carotenoids by HPLC

The carotenoid profile was determined by reverse phase HPLC using an Ultimate 3000 Basic Automated System (Thermo Fisher Scientific Inc., Waltham, MA, USA) fitted with a diode arrangement detector. The chromatographic separation was performed on a Beckman Ultrasphere C18 250 × 4.6 mm silica column (5 μm dia. spheres). Elution was conducted using a gradient of methanol/acetonitrile (B: 35% 5 min, B: 35–10% 10 min, B: 10–35% 5 min, B: 35% 10 min) at a flow rate of 1 ml min^−1^, and peak detection was performed at 480 nm. The peaks derived from the samples were identified by comparison to the spectra and retention time from astaxanthin and β-carotene standards (Sigma-Aldrich). All other reagents were purchased from J.T. Baker (Center Valley, PA, USA) unless otherwise specified.

### RNA extraction and cDNA synthesis

Broth samples (1.5 ml) were centrifuged and lyophilized, then RNA was extracted from 50 to 100 mg lyophilized solid. In brief, 5 mg glass beads (213–300 µm, Sigma-Aldrich) and 1 ml TRIzol reagent (Ambion, Life Technologies, Carlsbad, CA, USA) were added to each solid sample and homogenized using a plastic pistil; the samples were then incubated for 5 min at 24 °C. Subsequent RNA isolation was performed according to the manufacturer’s TRIzol protocol. The RNA in the tube was dried in a laminar flow hood, solubilized in 50 μl sterile RNase-free water, and quantified at 260 nm using a UV–Vis spectrophotometer (NanoDrop 2000c, Thermo Fisher Scientific). Samples were stored at − 20 °C until use. cDNA synthesis was performed using SuperScript^®^ III Reverse Transcriptase (Invitrogen, Carlsbad, CA, USA). The reaction mixture (20 μl) contained: 1 μl oligo (dT), 1 μl total RNA (5 μg μl^−1^), 1 μl of 10 mM dNTP mix, 4 μl of 5× First Strand Buffer, 1 μl DTT (0.1 M), 1 μl SuperScript III RT (200 units μl^−1^), and 11 μl sterile RNase-free water.

### Expression of *asy* and *aox*

The *asy* gene encodes a bifunctional P450 monooxygenase that catalyzes the oxidation of carbons 3 and 4 of β-carotene to give astaxanthin [36, 37]. qPCR for *asy* and *aox* was performed on a 7500 Real-Time PCR System using TaqMan^®^ probes designed by Applied Biosystems (Foster City, CA, USA) from the respective *P. rhodozyma* NRRL-Y-10922 genes (GenBank Accession Nos. KY967369, KY967370, and KY967371). The 10 μl reaction volume consisted of 2 μl cDNA (100 ng), 2 μl nuclease-free water, 5 μl 2 × TaqMan^®^ Universal PCR Master Mix, and 1 μl TaqMan^®^ 20 × probe. The amplification conditions were: 95 °C (10 min), 40 cycles at 95 °C (15 s), and 60 °C (1 min). The threshold cycle (Ct) was determined using the Sequence Detection v1.2.2 program. The CT values were normalized to the respective values of the *adki* endogenous gene from *P. rhodozyma* and were expressed as a function of a 12-h sample of the culture +Cu using the 2^−ΔΔCT^ method [38]. Each determination was carried out in triplicate. Reactants and laboratory equipment were from Applied Biosystems.

### Statistical analysis

ANOVA (*α *=* 0.05*) and Tukey test were performed using Minitab^®^ program 17.3.1, 2013 (Minitab, Inc., State College, PA, USA).

## Results

### Effects of copper deficiency on growth and respiration

**Fig. 1:**
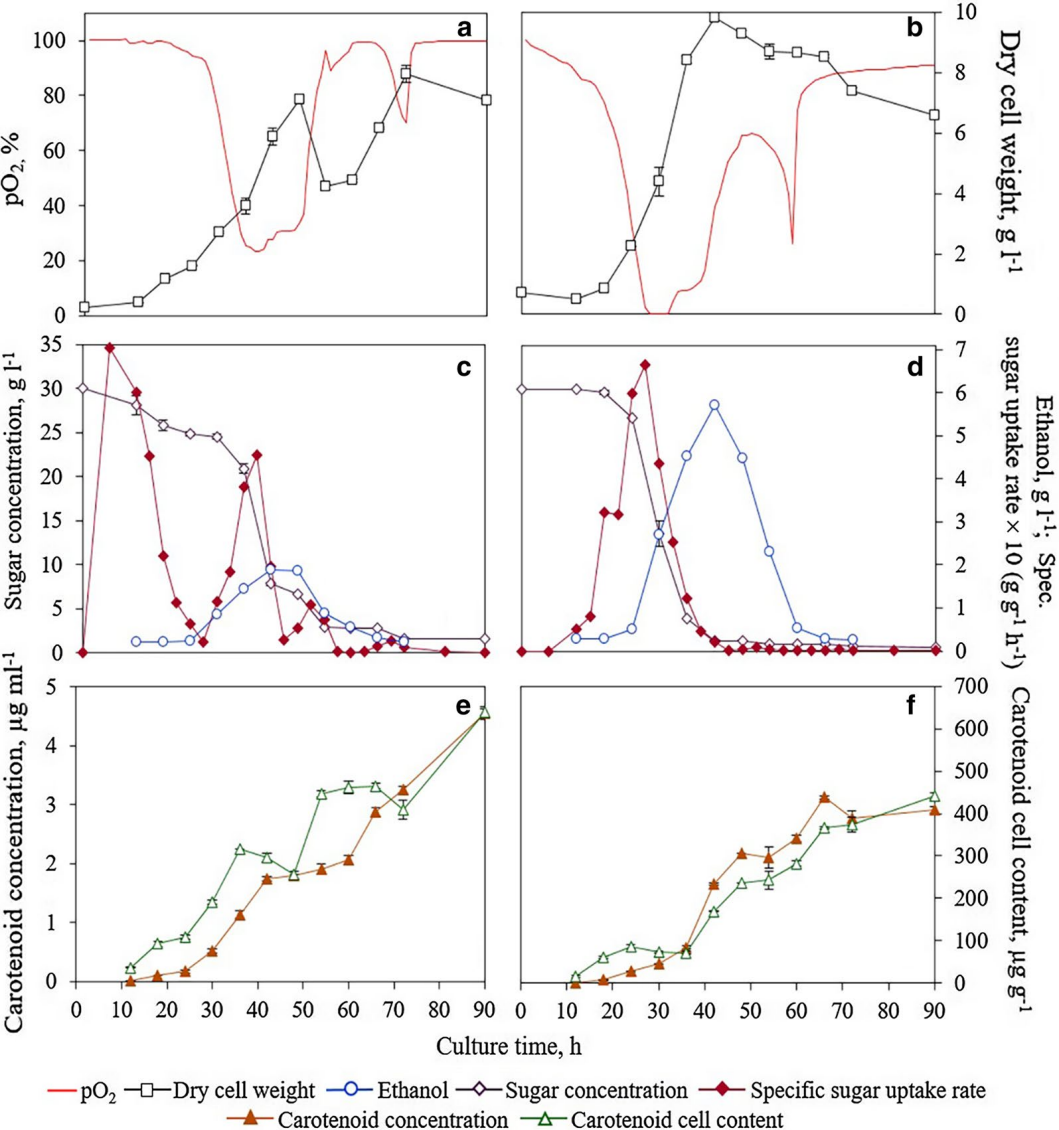
Development of culture parameters for *P. rhodozyma* grown in synthetic medium with (+ Cu culture) and without Cu^+2^ (− Cu culture). **a**, **c**, **e** correspond to the − Cu culture, whereas **b**, **d**, **f** correspond to the + Cu culture. Culture conditions: 1 vvm, 700 rpm, 22 °C, pH 4.5. Each determination was carried out in triplicate; error bars correspond to the standard deviation

During the accelerated growth period (18–42 h), *P. rhodozyma* grew 42% faster (μ avg 0.37 h^−1^) in the presence (+Cu), as compared with the absence (−Cu) of copper (μ avg 0.26 h^−1^). In addition, the maximum growth was 22.5% greater in the culture +Cu (9.8 vs. 8.0 g l^−1^) and was reached earlier (42 h) in comparison with the culture −Cu (72 h) as shown in Fig. 1a, b. Thus, the deficiency of copper negatively affected both the growth rate and the maximum biomass concentration attained. Similarly, the lack of copper slowed the oxygen uptake rate from the beginning of the culture −Cu (0–18 h). Subsequently, this was also evidenced during the fast growth period by the somewhat less pronounced fall of dissolved oxygen tension (pO_2_) in the culture −Cu (Fig. 1a, slope − 8.26 between 28 and 36 h) in comparison with the culture +Cu (Fig. 1b, slope − 10.1 between 21 and 26 h). In addition, the pO_2_ did not fall below 23.4% saturation (39 h) in the culture −Cu, whereas values close to zero were apparent for a few hours (27–31 h) in the culture +Cu. A diminished yield (Y_X/S_) of *P. rhodozyma* grown in the absence of copper has also been reported, indicating a low efficiency of energy conversion and most likely lower ATP production as well [12].

### Effects of copper deficiency on sugar consumption

It is evident that the absence of copper was a hindrance to the utilization of sugar (Fig. 1c, d). In fact, sugar exhaustion occurred much later in the culture −Cu (at 72 h, 1.7 g l^−1^) compared to the culture +Cu (at 42 h, 1.2 g l^−1^). Furthermore, the culture −Cu exhibited time periods where sugar consumption temporarily slowed or stopped (around 12–30, 42–48 and 54–66 h). Also, within each one of these periods, significant drops in the specific sugar uptake rate can be seen. Figure 1c shows a sizeable decrease in the specific sugar uptake rate between 12 and 27 h (0.6 and 0.025 g sugar g cells^−1^ h^−1^, respectively), despite the presence of sugars at levels around 25 g l^−1^. Conversely, shortly after the beginning of the accumulation of ethanol (24 h), the cells resumed sugar consumption at 27 h to a maximum at 39 h (0.46 g sugar g cells^−1^ h^−1^), almost when the accumulation of ethanol was coming to an end (Fig. 1c). A second decrease in specific sugar uptake rate took place from 39 to 45 h (0.46 to 0.029 g sugar g cells^−1^ h^−1^, respectively). This decrease was largely associated with the rapid decrease of sugar concentration within the period, but also with the insufficient sugar levels (about 7–8 g l^−1^ between 42 and 48 h) to sustain alcoholic fermentation under the existing aerobic conditions (note the steady ethanol levels between 42 and 48 h). Following a short term recovery (45–51 h), a third yet slight decrease in specific sugar uptake rate ensued between 51 and 57 h (0.11–0.003 g sugar g cells^−1^ h^−1^, respectively). This was visibly associated with the time frame in which ethanol was being actively utilized. The consumption of sugars again ceased temporarily between 54 and 66 h, but was briefly reinstated when ethanol was about to be exhausted. Altogether the previous data, denote that all three prominent falls in the specific sugar uptake cited above, evolved from corresponding redox imbalances, i.e., from an impaired capability to oxidize NADH (reduced nicotinamide adenine dinucleotide) into NAD ^+^ (the oxidized form).

Moreover, the culture +Cu displayed more continuous sugar consumption from the beginning and until the exhaustion of sugar in the broth (42 h). Figure 1d shows, following the exit of the lag phase, a rise in specific sugar uptake rate to maximum 0.66 g sugar g cells-1 h-1 at 27 h. Next a drop in specific sugar uptake rate took place between 27 and 42 h (0.66 and 0.021 g sugar g cells^−1^ h^−1^, respectively). This sole decline was closely linked with the rapid decrease of sugar concentration within the period.

### Effects of copper deficiency on ethanol production

Figure 1c, d show a bell-shaped behavior of ethanol concentration in both cultures. This is in accord with the typical production of ethanol during the initial hours of growth of several other yeasts in culture, and its subsequent reassimilation/consumption upon sugar exhaustion. Ethanol accumulation began at 24 h and attained maximal levels at 42 h. In the culture +Cu, ethanol began to accumulate shortly after the pO_2_ decreased below 25% saturation (25–40 h), and coincident with a rapid sugar consumption (Fig. 1d). On the contrary, in the culture −Cu the beginning of ethanol accumulation was not related with low pO_2_, but with the decrease in specific sugar uptake rate, i.e., when it was approaching to levels close to zero (24 h). It is striking that *P. rhodozyma*, when grown in –Cu, began the synthesis of ethanol unpredictably from 24 h, despite the high prevailing pO_2_ (94% saturation). Thus, *P. rhodozyma* exhibits aerobic fermentation when grown under copper deficiency. The maximum level of ethanol achieved was markedly lower in the culture −Cu (1.9 g l^−1^) than in the culture +Cu (5.7 g l^−1^), apparently as a result of the greater average pO_2_ prevalent in the former condition. In effect, ethanol production by *P. rhodozyma* was inhibited by the lack of copper. Similarly, the average rate of ethanol production was lower in the culture −Cu (0.09 g l^−1^ h^−1^ vs. 0.29 g l^−1^ h^−1^).

### Effects of copper deficiency on ethanol reassimilation

The reassimilation of ethanol started at 48 h in the culture −Cu, although some sugar was still available in the culture broth (6.7 g l^−1^). In contrast, the utilization of ethanol began at 42 h in the culture +Cu, shortly after sugar exhaustion (40 h), and while pO_2_ was pronouncedly increasing up to 65.9% at 50 h (Fig. 1d). The activation of ethanol reuse in the latter culture caused a steep decline in pO_2_ (52–60 h); on the contrary, when the available ethanol was exhausted, pO_2_ again rapidly increased to values close to 100%. The average ethanol consumption rate was markedly faster in the culture +Cu (0.29 g l^−1^ h^−1^, 42–60 h) compared with the culture −Cu (0.09 g l^−1^ h^−1^, 48–66 h). Considering that ethanol metabolism demands an active respiration; the lower rate of ethanol consumption when copper was absent is also consistent with a respiratory impairment in the yeast cells grown under such conditions (48–66 h). The culture −Cu, unlike the culture +Cu, showed only a marginal decrease of pO_2_ (pO_2_ > 88.9%, 54–55 h) during ethanol reassimilation, which also points out the inferior respiratory activity of the yeast cells lacking copper.

### Effects on carotenoid accumulation

Figure 1e, f show the carotenoid content of yeast cells grown in −Cu and +Cu, respectively, along with the development of carotenoid concentration. Even during the early growth period (30–36 h), the cellular carotenoid content was already higher in the yeast cells grown in −Cu (α = 0.05; ANOVA). By the end of fermentation (90 h), the cellular content of carotenoids was markedly higher in the culture −Cu (641 μg g cells^−1^) compared with the culture +Cu (442 μg g cells^−1^). A Tukey test applied to the two complete sets of data (−Cu and +Cu) showed that the cellular content of carotenoids was enhanced by lack of copper (p < 0.05).

The concentration of carotenoids was also significantly greater by the end of the fermentation (90 h) in the culture −Cu (4.6 vs. 2.9 μg ml^−1^). However, in this case three stages could be distinguished overall: i) in the first stage (i.e., 36 h) the carotenoid concentration was slightly greater in the culture −Cu than in the culture +Cu; ii) in the second stage (48–66 h), the carotenoid concentration was greater in the culture +Cu in comparison with the culture –Cu; and iii) in the third stage (66–90 h), the carotenoid concentration was again greater in the culture −Cu. In the first stage, the lack of copper encouraged a rapid accumulation of carotenoids in the yeast cells (12–36 h), which prevailed over the slower growth of the yeast, as compared to the culture +Cu. In the second stage, the greater concentration of biomass and greater amount of assimilated ethanol in the culture +Cu as a whole contributed to a greater concentration of carotenoids in the culture +Cu (between 8 and 18%), as compared to the culture −Cu. In the third phase toward the end of the fermentation, a marked accumulation of carotenoids could be observed in the culture −Cu, presumably associated with the use of an unknown carbon source, as suggested by the fall in pO_2_ to a minimum 70% around 66–72 h. In contrast, the culture +Cu showed a small decrease of the concentration of carotenoids (66–72 h), which apparently was linked to the decrease in biomass that occurred after exhaustion of the main sources of carbon and energy (sugar and ethanol).

A positive slope of intracellular astaxanthin accrual by *P. rhodozyma* is usually an outstanding gauge of environmental conditions that impose stress on the yeast cells. Substantial accruals of specific intracellular carotenoids were developed within 12–18, 24–36, 48–54, and 72–90 h, in the culture –Cu. Concurrently, substantial increases of carotenoid concentration occurred from 24 to 42 h and 60–90 h. In particular, carotenoid accrual during the early growth period appeared positively linked with the increase in biomass. The culture +Cu also exhibited significant accrual of intracellular carotenoids during several time periods (12–18, 36–48, 54–66, and 72–90 h). Additionally, pronounced increases of carotenoid concentration could be seen from 36–48 and 54–66 h, and a much slighter rise from 18 to 36 h. However, in the culture +Cu, the synthesis of carotenoids, to a large extent, was not associated with biomass accumulation over time. The carotenoids accumulation during the early hours, regardless of whether the cells were grown in −Cu or +Cu, evidence that the relatively high pO_2_ prevailing already exposed the yeast cells to significant oxidative stress [see also 39]. However, the more positive slope of intracellular carotenoids accumulation in the culture −Cu suggested that another additional factor contributed to such oxidative stress. The greater *aox* expression in the culture –Cu implied that a redox imbalance contributed to the surplus carotenoids accrual, as discussed in further detail below. Moreover, it is relevant that ethanol reassimilation starting from 42 h (+Cu) and 48 (−Cu), encouraged the intracellular accrual of astaxanthin in both cultures. Given that ethanol supplies more reducing equivalents per C mol that glucose, it is thus conceivable that a redox imbalance triggered the intracellular astaxanthin accrual during ethanol assimilation.

**Fig. 2:**
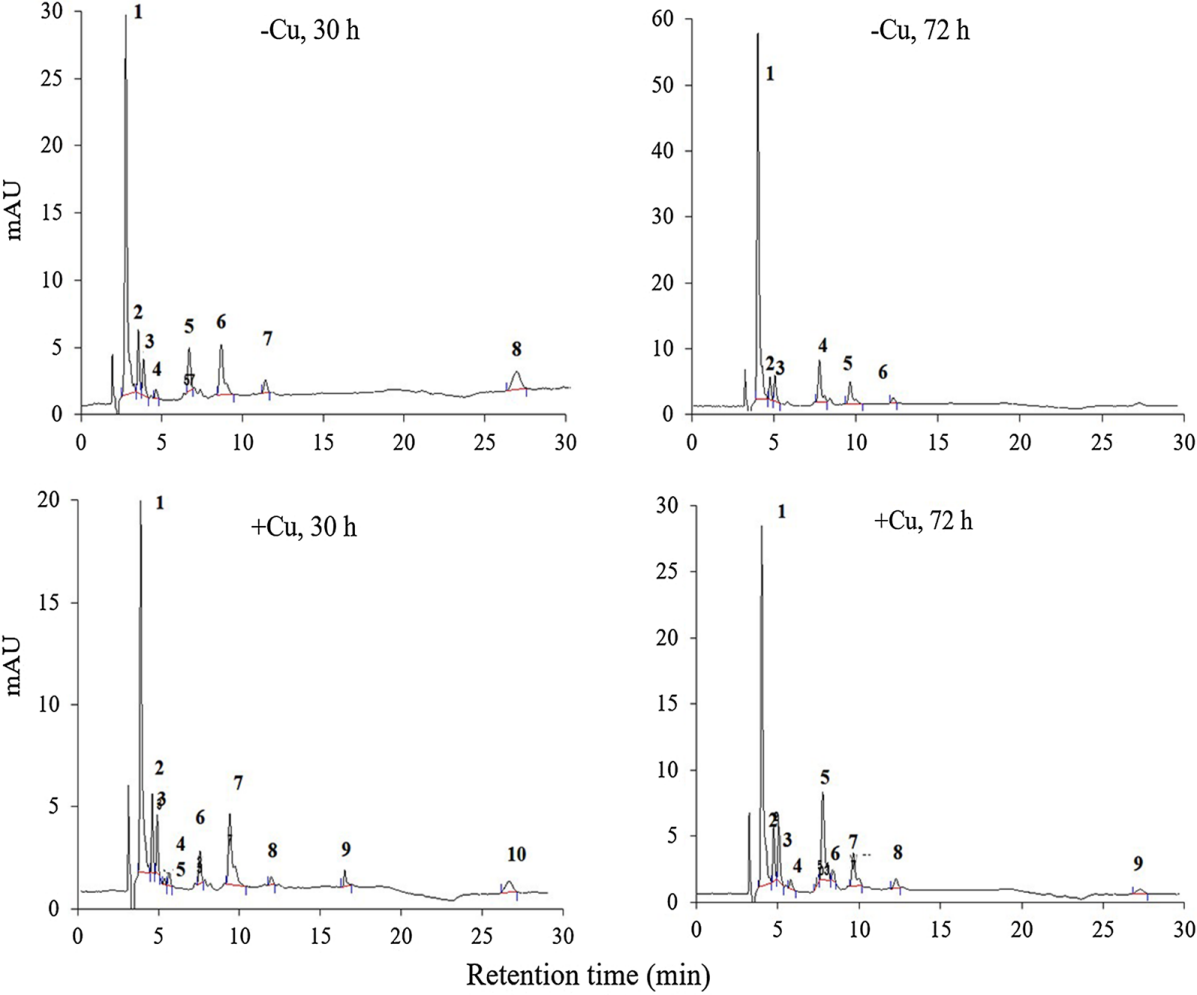
Reverse phase HPLC chromatogram showing the carotenoids extracted from *P. rhodozyma* cells grown with (+ Cu culture) and without Cu^+2^ (− Cu culture). The yeast cells were recovered at 30 and 72 h, just at the onset of ethanol accumulation and after the exhaustion of ethanol, respectively. Peak 1 represents astaxanthin in all instances. Peak 8 in − Cu, 30 h; peak 10 in + Cu, 30 h and peak 9 in + Cu, 72 h correspond to β-carotene. All other peaks correspond to intermediary unknown carotenoids. Peak detection wavelength was at 480 nm using a diode arrangement detector

Figure 2 shows the profile of carotenoids of *P. rhodozyma* cells grown in the presence and absence of copper at 30 and 72 h: i.e., from cells recovered just at the onset of ethanol accumulation and after the exhaustion of ethanol. Carotenoids extracted from yeast cells grown in −Cu contained a higher proportion of astaxanthin (76% at 72 h) in comparison with those grown in +Cu (56%). A higher proportion astaxanthin has been known to be pointer of enhanced cellular oxidative stress [12]. In the culture −Cu (Table 1), the fraction of astaxanthin increased with time whereas the proportion of β-carotene decreased. In contrast, the fraction of astaxanthin remained relatively stable in the culture +Cu and the decrease in the fraction of β-carotene was less pronounced over time.

**Table 1:** Proportion of astaxanthin and β-carotene in pigments extracted from *P. rhodozyma* cells grown under −Cu and +Cu

Cell sampling time (h)	30	66	72	Culture condition
Astaxanthin %	54 ± 3	73 ± 1	76 ± 1	−Cu
	57 ± 2	50 ± 0.5	56 ± 1	+Cu
β-Carotene %	17 ± 2	5 ± 1	2 ± 0.5	−Cu
	9 ± 1	6 ± 1	6 ± 1	+Cu

### Effects of copper deficiency on the expression of *asy* and *aox*

**Fig. 3:**
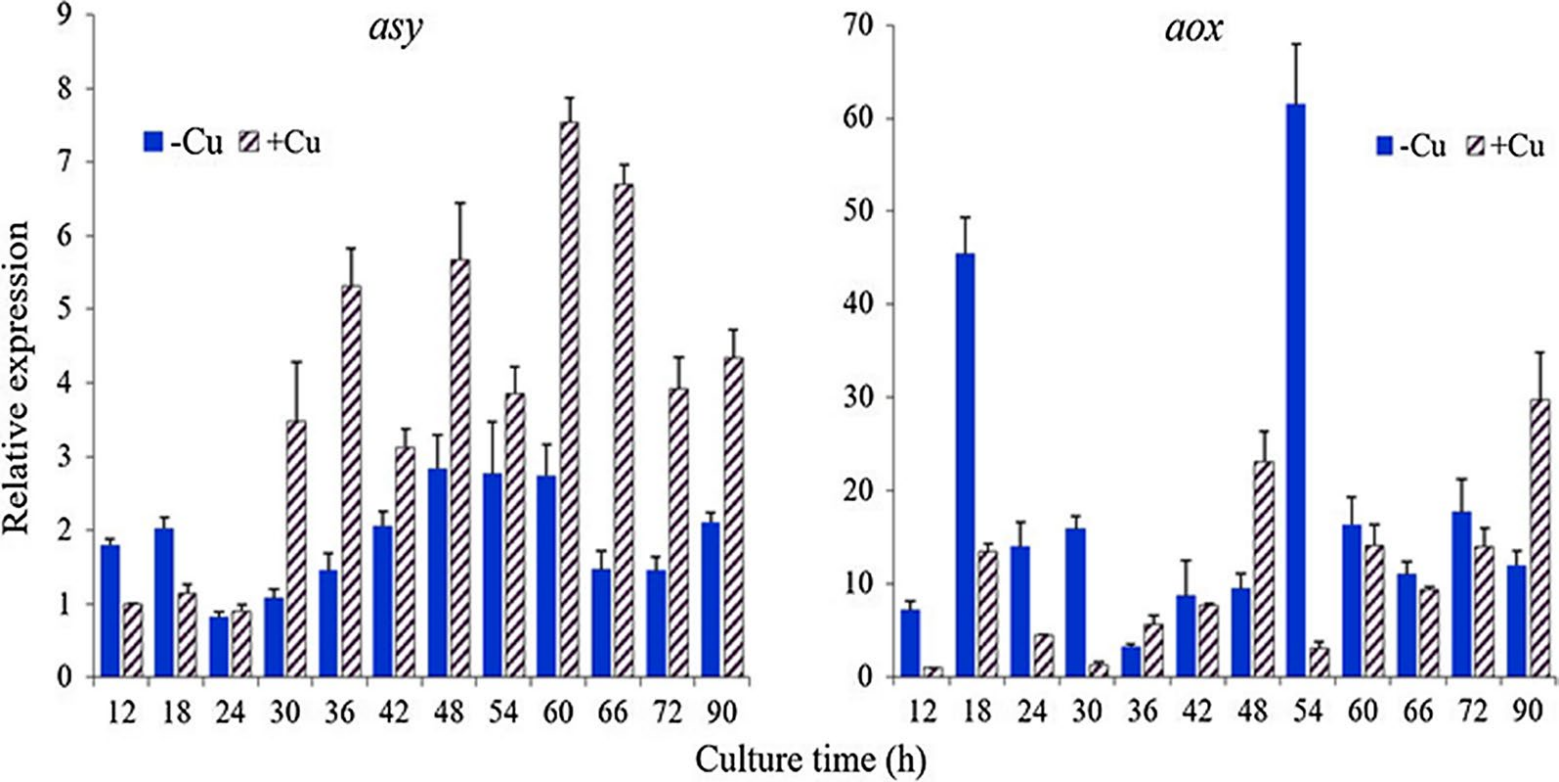
Relative expression levels of *aox* and *asy* in cells of *P. rhodozyma* grown in the cultures − Cu and +Cu. Expression of *asy* and *aox* both, are given as relative values to the expression at 12 h in the culture +Cu, which was arbitrarily set as 1. *Adki* was used as an internal control for normalization. Each determination was performed in triplicate; error bars correspond to standard deviations

Figure 3 shows that at the early growth period (12–18 h), the expression of *asy* was 1.6-fold greater in the culture −Cu than in the culture +Cu, whereas the pO_2_ was close to 100% saturation in the culture −Cu although somewhat lower in the culture +Cu (77%, 18 h, Fig. 1). However, after 24 h and through the end of the cultivation period, the expression of *asy* was always higher in +Cu as compared to −Cu, albeit in a different proportion depending on the culture age. Moreover, the expression of *aox* was almost always greater in the culture −Cu compared to the culture +Cu at each comparable time (Fig. 3). The only exception was observed at 48 h, which ensued shortly after the beginning of ethanol utilization (42–48 h) in the culture +Cu, whereas in −Cu the ethanol reassimilation lagged behind relative to the culture +Cu; i.e., the uptake of ethanol began somewhat later in −Cu (48–54 h).

In particular, marked increases of *aox* expression can be seen between 12 and 18 h (6.3-fold), and from 48 to 54 h (6.5-fold) in the culture −Cu. The first raise in *aox* expression (12–18 h) was concomitant with substantial accrual of carotenoids (Fig. 1e), and made us assume that the absence of copper led successively to impaired oxygen uptake, redox imbalance, and oxidative stress on the yeast cells. Furthermore, as shown in Fig. 1a, c, a notably decline in specific sugar uptake rate can be seen during this early growth period (< 24 h), followed by a mild pO_2_ drop (dO_2_/dt 0.49 g l^−1^ h^−1^). The induction of ethanol production shortly thereafter (24–36 h), restored the consumption of sugar (Fig. 1c), and carotenoid synthesis (Fig. 1e), despite the fact that the drop in pO_2_ intensified even more (dO_2_/dt 3.3 g l^−1^ h^−1^). Overall, the preceding data discernably indicate that a progressive redox imbalance during the early growth period, which arose from an impaired respiration in the cells grown in absence of copper, was able to trigger both the marked rise of *aox* expression as well as the aerobic fermentation. Evidently, the cited redox imbalance was eased by upgrading the conversion of NADH to NAD^+^(to restore the needed supply of NAD^+^ for glycolysis and growth), when alcoholic fermentation set off after 24 h. The diminished expression of *aox* at 36 h; i.e., 20% of the expression level seen at 24 h, and only 7% of the maximum expression detected at 18 h, indicates that once NADH reoxidation was restored through aerobic fermentation, the electron transport through AOX (24–36 h) became needless. In culture −Cu, the second substantial increase in the expression of *aox* (6.5-fold) occurred while ethanol was being consumed (48–54 h) by the yeast cells, and was accompanied by a rapid accrual of intracellular carotenoids. Similarly, in the culture +Cu, the expression of *aox* was also activated (3-fold) at the beginning of ethanol reassimilation (36–48 h) and was also associated with a rapid accrual of carotenoids. Notably, the rise in expression was more prominent in the culture −Cu (6.5-fold), despite the relatively small amount of ethanol available and the lower rate of ethanol consumption in comparison with the culture +Cu. In summary, the reassimilation of ethanol encouraged carotenoid synthesis and the expression of *aox* in both cultures. Nevertheless, the ethanol reassimilation together with the absence of copper stimulated the expression of *aox* to a much greater degree than the reassimilation of ethanol in presence of copper.

## Discussion

### Global effects of copper deficiency

Copper deficiency in *P. rhodozyma* had a detrimental effect on all of the following: (i) the maximum growth rate and end biomass; (ii) the oxygen uptake rate; (iii) the maximum ethanol concentration reached; (iv) the ethanol production rate; (v) the ethanol consumption rate; and (vi) the specific sugar uptake rate. Conversely, the lack of copper encouraged alcoholic fermentation under aerobic conditions, and subsequently, ethanol reassimilation despite the presence of sugars in the culture broth. In addition, the lack of copper had a favorable effect on (i) the carotenoid content of the yeast cells; (ii) the final concentration of carotenoids; (iii) the proportion of astaxanthin in the carotenoids produced; and (iv) the expression of *aox*. The lack of copper had a positive effect on *asy* expression at the beginning of the culture but had a negative effect after 24 h. In both conditions tested, a pronounced *aox* expression occurred at the early growth period (12–18 h), when pO_2_ was close to saturation, as well as while cells were consuming ethanol (48 and 54 h), but especially in the culture −Cu.

### Effects of copper deficiency on oxygen consumption

Mitochondria are primary sites of oxygen consumption in eukaryotic cells. As such, mitochondria actively respiring oxygen generate a gradient of pO_2_ with their environment. Hence, the mitochondria of actively growing cells are usually exposed to, and function at, considerably lower pO_2_ than the average levels prevailing in the culture broth upon saturation with air [40, 41]. For example, the vigorous oxygen consumption occurring between 28 and 32 h in the culture +Cu caused a fall of pO_2_ to levels close to zero in the broth, despite the continuous air supply. In contrast, the culture −Cu exhibited largely greater pO_2_ levels as a result of the slower oxygen uptake elicited by the absence of copper. This is consistent with previous reports showing that copper deficiency, in several cell types, diminishes; (i) cellular content of CcO; (ii) CcO activity; and (iii) mitochondrial respiration [22, 28, 42–45]. Furthermore, it can be expected that the mitochondrial level of pO_2_ is set, in vivo, from the balance between delivery and oxygen consumption within mitochondria. Oxygen delivery is largely determined by the pO_2_ in the air inside the bioreactor, the air flow rate, and the stirring rate, whereas consumption depends mostly on mitochondrial cellular respiratory activity. Thus, under a fixed set of operating conditions, the mitochondrial pO_2_ is to a great extent dependent on mitochondrial cellular respiratory activity, i.e., changes of oxygen uptake rate lead to corresponding changes of mitochondrial pO_2_. That is, vigorous oxygen consumption (+Cu) lessens the mitochondrial pO_2_, whereas slow oxygen consumption (−Cu) invariably leads to greater pO_2_. Accordingly, and consistent with the measured pO_2_ levels in the broth, we can assume that mitochondria of cells grown in absence of copper were exposed, on average, to higher pO_2_ than those grown in +Cu, and from there exposed to more severe oxidative conditions.

### Copper deficiency affects the redox balance

During cell respiratory growth, electrons from both cytosolic and mitochondrial NADH are transferred to oxygen via the ubiquinone pool (Q/QH_2_). Electrons from QH_2_, which is an obligatory component of the mitochondrial electron transport chain, can be funneled either through the main mitochondrial respiratory chain or alternatively through an alternative oxidase, AOX [26, 27]. In many cell types, it has been long known that respiratory activity modulates the redox state of the NADH/NAD^+^ couple in vivo [46–48]. Namely, when respiration slows, the NADH/NAD^+^ ratio increases; whereas an active respiration diminishes this ratio through rapid NADH conversion to NAD^+^ [22, 41, 49]. The relatively slow oxygen uptake rate of the cells grown in −Cu suggested that the lack of copper raises the proportion of electron carriers in a reduced state (e.g., NADH and QH_2_) bound to proteins; e.g., NADH dependent dehydrogenases and complex III [50–52]. In contrast, an active respiration (+Cu) may lead to a more oxidized state of such electron carriers. Thus, we should have to assume that electron carriers in the cells grown in −Cu were, on average, more reduced owing to the slower respiration than those of cells grown in +Cu. In other words, fluctuations of respiratory activity, besides causing dynamic changes of pO_2_, presumably cause dynamic changes in the redox state of the redox cofactors as well.

### Copper deficiency induces carotenoid synthesis

The largely greater carotenoid content of the yeast cells grown in −Cu throughout the fermentation, as well as the higher concentration of intracellular carotenoids by the end of fermentation, indicate that when grown in −Cu, *P. rhodozyma* cells were confronted with greater oxidative stress than those grown in +Cu. Consequently, we inferred that the lack of copper encouraged the formation of O_2_^·−^/H_2_O_2_, requiring the cells to protect themselves through accrual of carotenoids. Moreover, as copper deficiency results in higher average pO_2_ in comparison with the culture +Cu, it is conceivable that the higher pO_2_ levels are linked with an augmented production of O_2_^·−^/H_2_O_2_, as well as the intracellular carotenoids. In agreement with this, substantial evidence has shown that high pO_2_ levels caused by CcO dysfunction commonly prompt mitochondrial ROS production in other cell types [20, 41, 44, 45, 53]. Moreover, in yeasts other than *P. rhodozyma*, NADH levels and the rate of O_2_^·−^/H_2_O_2_ production both depend on mitochondrial respiratory activity as reviewed by Barros et al. [54]; however, the precise mechanism involved, in vivo, has remained unclear [51, 52]. As a whole, the in vivo data mentioned above are consistent with what has been well established for isolated mitochondria, wherein the rate of production of O_2_^·−^, the primary ROS (which can be enzymatically or spontaneously converted to H_2_O_2_), is positively dependent on both, pO_2_ [50, 53] and the reduction state of electron carriers (e.g., NADH) bound to proteins [27, 41, 50–52, 55–58]. The rapid accumulation of intracellular carotenoids between 48 and 54 h, and the maximum expression levels of both *asy* and *aox* at 54 h in cells grown in –Cu further reinforce this inference (see further discussion below). Thus, *P. rhodozyma* cells, when grown in absence of copper, markedly activated the synthesis of astaxanthin owing to the high pO_2_ prevailing (Fig. 1a) and, apparently, to the uppermost degree of reduction of the cofactors bound to proteins of the respiratory chain, in contrast to those in culture +Cu. These events to all appearances arose from the diminished oxygen uptake rate resulting from the lack of copper. Several other particular events that took place along with alcoholic fermentation also serve to underwrite the decisive role of redox imbalances behind the induction not only of carotenoid synthesis and *aox* expression, but also of fermentation regardless if aerobic or anaerobic. In the following sections, we argue in some detail on the accumulation of carotenoids during the time frames of ethanol production and ethanol reassimilation by *P. rhodozyma*.

### A decoupled redox balance explains the induction of both, aerobic fermentation and *aox* expression

The three declines in specific sugar uptake rate (Fig. 1c) seen in the culture −Cu are strikingly informative to appreciate the relevance of redox imbalances on the adaptive cellular response. Here we analyze the first two declines, and later on the latter. The notable decrease in specific sugar uptake rate between 12 and 27 h (0.6 and 0.025 g sugar g cells^−1^ h^−1^, respectively), suggested a progressive cellular impediment to metabolize the available sugar as carbon source. Nonetheless, the cells resumed sugar consumption at 27 h, shortly after the beginning of ethanol build up. The second decrease in specific sugar uptake rate began when the accumulation of ethanol was coming to an end (39 h). It is well known that alcoholic fermentation promotes NADH re-oxidation, whereas deactivation of fermentation lessens NADH turnover. Accordingly, the above data strongly suggested that the decreases in specific sugar uptake rate came associated with redox imbalances caused by the lack of copper, i.e., as a result of an impaired re-oxidation of NADH. During the early growth (18 h), the yeast cells grown in −Cu, showed greater expression of *aox* (3.4-fold) and *asy* (2-fold), along with greater intracellular accumulation of carotenoids (1.5-fold) compared with those grown in +Cu. The greater expression of *aox* and *asy*, and slightly thereafter the greater intracellular carotenoid accumulation (e.g., 24–36 h), as argued previously, are in accord with the interpretation that the lack of copper impaired respiration and thence the re-oxidation of NADH. This is also in accord with the generally accepted role of AOX; i.e., to dissipate the energy of reducing equivalents into heat to safeguard cells against severe oxidative stress [25, 28, 59, 60]. When the main respiratory chain is hampered, AOX activity encourages mitochondrial NADH reoxidation by providing an alternative pathway for the passage of electrons from QH_2_ to oxygen. Therefore, it is apparent that an impaired NADH reoxidation with somewhat higher pO_2_, both encouraged *aox* expression. Furthermore, in cells with impaired mitochondrial respiration, a redox imbalance could easily spread to the cytoplasm [46, 61]. Under such circumstances, fermentative metabolism became a necessity for yeast cells, despite the prevalent aerobic conditions. The onset of the aerobic fermentation (24 h) highlights the need to stimulate cytosolic NADH turnover, sugar consumption, and ATP synthesis while mitochondrial function was hampered in the cells grown in −Cu (Fig. 1c, see also [27, 61, 62]). The significant accumulation of intracellular and volumetric carotenoids seen from 24 to 36 h suggested that despite the onset of aerobic fermentation, copper deficiency still elicited some stress on the yeast cells. Moreover, the decline of *aox* and *asy* expression from 18 to 24 h (Fig. 3) points out that the induction of aerobic fermentation by promoting NADH reoxidation diminished the necessity of the mitochondrial AOX activity for NADH turnover. Importantly, *aox* expression and aerobic fermentation were both evidently triggered by a redox imbalance in the *P. rhodozyma* cells grown in absence of copper. It is also relevant that ethanol concentration became stable between 42 and 48 h (−Cu), despite the availability of sugars at levels between 6.7 and 7.9 g l^−1^. This denotes that the low sugar levels were insufficient to sustain aerobic fermentation, so that, the resultant scanty NADH turnover became once more a limiting factor to keep sugar metabolism going.

### A decoupled redox balance also explains the induction of anaerobic fermentation

Cells grown in presence of copper started alcoholic fermentation as usual; that is, when they were in the anaerobic growth phase (24–30 h). Then, low oxygen levels triggered anaerobic fermentation, whereas copper deficiency triggered aerobic fermentation. However, the two conditions impair oxygen consumption, as suggested by the small pO_2_ increase occurring from 32 to 36 h in the former condition. Hence, low oxygen levels should lead to a redox imbalance as well. In agreement with this, increasing NADH/NAD^+^ ratios have been reported, in vivo, in different cell types exposed to graded hypoxia [49]. Thus, we could assume that more reduced electron carriers, of themselves, resulted in increased O_2_^·−^/H_2_O_2_ generation that served to signal the existence of hypoxic conditions [63–66]. The small increases of *aox* and *asy* expression from 30 to 36 h are also in agreement with the above interpretation. This implies that alcoholic fermentation, regardless of whether aerobic (−Cu) or anaerobic (+Cu), as well as the expression of *aox*, and astaxanthin synthesis, albeit under some distinctive circumstances, all are triggered following redox imbalances. Accordingly, alcoholic fermentation, mitochondrial respiration, and AOX function all represent crucial components acting in concert to preserve overall redox homeostasis, and prevent severe oxidative stress on the yeast cells. It is well established that under hypoxic conditions, NADH can be oxidized to NAD^+^ via another critical pathway, namely glycerol formation [67, 68]. Other mechanisms of NADH reoxidation, such as uncoupling proteins (mitochondrial), may also function toward this end [27, 69].

The underlying biological cause that triggers aerobic fermentation has long been debated. However, despite considerable research, a definitive understanding of the mechanism involved has yet to be achieved. Strikingly, recent studies with *Saccharomyces cerevisiae* also show the crucial role of the NADH/NAD^+^ couple redox state to trigger fermentation under other conditions [70–72]. This may imply that redox imbalances trigger alcoholic fermentation under diverse conditions, not only under anaerobic conditions. As the biochemistry of the intermediary metabolism, including the NADH/NAD^+^ couple, is conserved from yeasts to humans [73, 74], it is discernible that the mechanism disclosed in this study for *P. rhodozyma*, might also be operative for triggering fermentation under the differing conditions reported for other yeasts and cell types [62, 67, 70, 73, 75–83]. In this sense, given that the short-term Crabtree effect and the Warburg effect (in tumors) both constitute part of an overall mechanism to redirect the metabolic flow toward fermentation [84], the mechanism described above for *P. rhodozyma* might be relevant to explain these two effects as well.

### Copper deficiency with ethanol reassimilation bring about detrimental oxidative stress

The third and final decline in specific sugar uptake rate (Fig. 1c) observed in the culture −Cu was associated with the time period when there was rapid consumption of ethanol. Ethanol metabolism demands an active respiration, and yields more NADH per carbon equivalent compared to glucose [85]. Ethanol reassimilation increases both the supply of NADH to the respiratory chain as well as the NADH/NAD^+^ ratio in many cell types [86–89]. Accordingly, the beginning of ethanol utilization by *P. rhodozyma* when copper was absent, would cause a sudden redox imbalance due to an impaired reoxidation of NADH through the respiratory chain. This suggested that the third decline in specific sugar uptake rate was also associated with a redox imbalance, in this case caused by an active ethanol utilization. In addition, ethanol utilization by *P. rhodozyma* encouraged the intracellular accumulation of carotenoids in both cultures (−Cu and +Cu); for example, while ethanol was being re-assimilated [48–54 h in −Cu (Fig. 1e); 36–48 h in +Cu (Fig. 1f)]. Furthermore, these events occurred concomitant with substantial decreases in cellular biomass, especially under copper deficiency (Fig. 1a). Together, these events highlight the remarkable stress conditions that developed while ethanol was being used as carbon source, especially when the respiratory activity was hampered by lack of copper. The slower ethanol consumption rate by cells in −Cu (Fig. 1c), when compared with those in +Cu (Fig. 1d), also seems to be indicative of the remarkable redox imbalance prevailing under the former condition. A redox imbalance together with relatively high prevalent oxygen levels would be expected to foster O_2_^·−^/H_2_O_2_ generation [51, 53, 90] and successive intracellular astaxanthin accrual. This is in accord with the higher accumulation of carotenoids in the yeast cells grown in −Cu, as compared to the culture +Cu when the ethanol became exhausted at 66 h (464 vs. 366 μg carotenoids g cells^−1^). It was evident that even small amounts of ethanol consumption, when coincident with impaired respiratory activity (−Cu), entails conditions of substantial stress on the yeast cells. The higher proportion of astaxanthin in the culture −Cu (76 vs. 56%) is also consistent with this interpretation (Table 1). In addition, the pronounced *aox* expression that took place alongside ethanol reassimilation, regardless of the presence or absence of copper, is also consistent with our inference that ethanol reassimilation resulted in a severe redox imbalance and harsh oxidative stress on the yeast cells. The culture −Cu showed a substantial increase of *aox* expression (6.5-fold) shortly after the onset of ethanol reassimilation (48–54 h). This was of vital importance, as higher *aox* expression would serve the imperative need to minimize the surplus of reducing equivalents, and by doing so limit the rate of intracellular O_2_^·−^/H_2_O_2_ generation [29, 30]. In addition, the sharp drop of biomass (48–54 h) suggested a significant cell death that is consequent with above interpretation.

In a similar way, in the culture +Cu, the expression of *aox* was also activated (3-fold) during the reassimilation of ethanol (42–48 h). The higher respiratory capacity, evidenced by the higher average rate of ethanol consumption (+Cu = 0.29 vs. −Cu = 0.09 g l^−1^ h^−1^) and sudden pO_2_-drop (Fig. 1b), would ensure a faster electron transfer to reduce molecular oxygen to water. Correspondingly, the predictably less pronounced redox imbalance necessitated lower *aox* expression levels in contrast with the culture −Cu. Therefore, the synthesis of astaxanthin together with the intermittent expression of *aox* during the period of ethanol uptake (42–60 h) appeared to play a key role in avoiding a drastic decrease of biomass (cell death) in the culture +Cu. In concordance, other authors have found that ethanol induces astaxanthin synthesis by *P. rhodozyma* [91–93]. Moreover, increased ROS production constitutes a common effect of ethanol metabolism in other cell types beyond yeasts [86, 90, 94].

In summary, a slowed electron flow through the main respiratory chain (−Cu), as well as increased electron supply at the beginning of ethanol reassimilation, both constitute conditions that could disturb the balance of NADH/NAD^+^ in favor of NADH. Therefore, we propose that induction of *aox* expression, as well as *asy* expression and the subsequent carotenoid synthesis are all triggered through a mechanism involving increased generation of O2^·−^/H_2_O_2_, which arises from the existence of a redox imbalance [93].

### Inhibitors and mutations that impair respiration trigger the synthesis of astaxanthin

A number of *P. rhodozyma* mutants with impaired respiration grow slowly and are able to accumulate increased concentrations of carotenoids as compared to their parental wild-type strains [95, 96]. Nonetheless, the precise nature of these mutations has not yet been elucidated. Additionally, several respiratory inhibitors have been shown to encourage the synthesis of astaxanthin by *P. rhodozyma* [4, 5, 32, 97]. For example, antimycin A inhibits the reoxidation of QH_2_ through interfering with the Qi site of complex III of the respiratory chain, thereby hampering electron transfer downstream to cytochrome c. Antimycin A triggers the synthesis of astaxanthin and increases the astaxanthin/total carotenoid ratio, along with O_2_^·−^ generation from complex III as well [3, 32]. The induction of astaxanthin synthesis concurred along with a shift from cyanide-sensitive respiration (CcO) to cyanide insensitive respiration (AOX) [97]. These authors proposed that the activation of AOX was closely associated with the synthesis of astaxanthin. However, a subsequent study by Hoshino et al. [33] showed that *aox* deletion in fact increased the cellular content of astaxanthin, indicating that AOX is not essential for astaxanthin synthesis. In accordance with this latter study, our data suggested that the activation of *asy* and *aox* was rather a successive response to increasing oxidative stress (Fig. 3), wherein the *asy* expression was activated initially, following the *aox* expression only when oxidative stress was beyond a certain safe level.

### An analogous signaling mechanism accounts for the induction of carotenoid synthesis under varying conditions

**Fig. 4:**
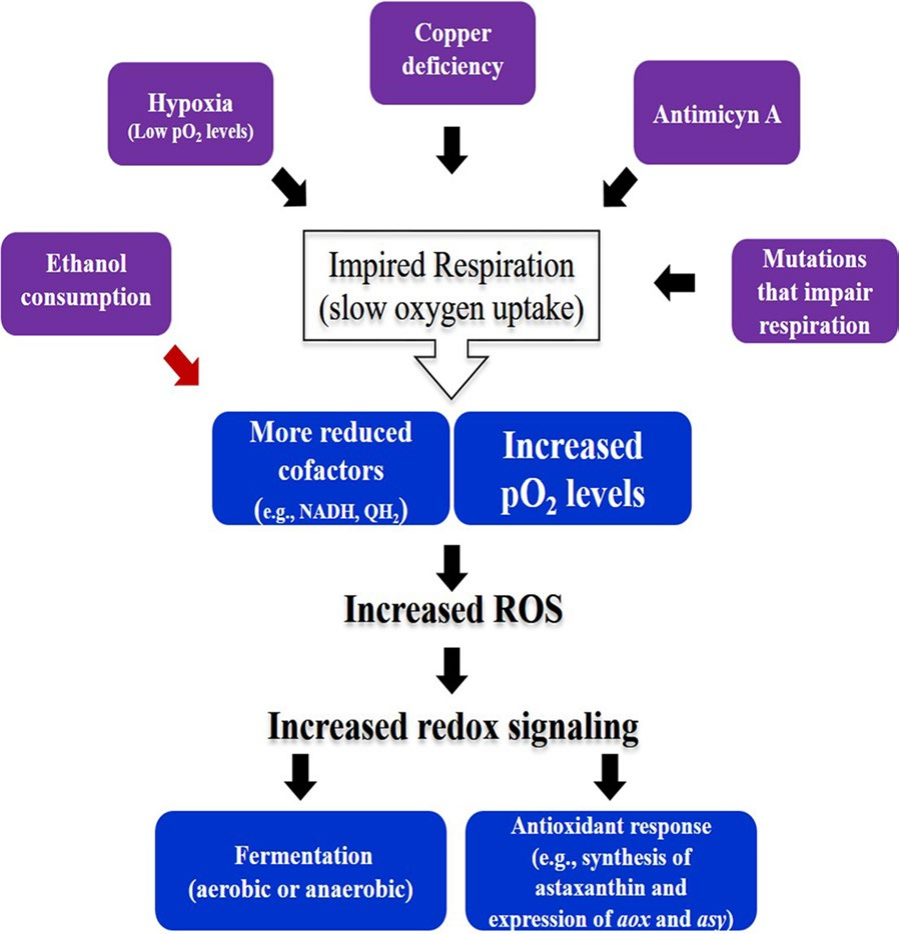
Proposed mechanism explaining how *P. rhodozyma* cells sense and develop an adaptive response to environmental cues, or to mutations in genes impairing respiration. The mechanism rationalizes the central role that redox imbalances play along with changes in mitochondrial pO_2_, to generate a dynamic redox signal that inform the yeast cells about changing environmental conditions and mutations. The dynamic redox signal serves to steadily tune an adaptive response. Copper deficiency, respiratory inhibitors (e.g., antimycin-A), low mitochondrial pO_2_ level and mutations impairing respiration, all are events leading to similar sequential outcomes, (i) slow oxygen uptake rate concurrently with slow NADH conversion to NAD^+^, (ii) increasing mitochondrial pO_2_ together with more reduced state of cofactors (NADH/NAD^+^ and QH_2_/Q couples) of the main electron transport chain, (iii) augmented O^2·−^/H_2_O_2_ formation as a result from the events ii, each one on its own, (iv) antioxidant response induction (e.g., astaxanthin synthesis and, asy and *aox* expression), and (v). induction of alcoholic fermentation (low oxygen levels triggers anaerobic fermentation, whereas copper deficiency triggers aerobic fermentation). Ethanol consumption at the diauxic shift leads to a NADH surplus as primary outcome of a faster NADH replenishment (red arrow, to emphasize that the primary source of the redox imbalance is not an impaired respiration at first), and secondly, to an impaired oxygen consumption as a result of NAD^+^ deficit (for clarity not shown in the figure). Successively these events lead to similar outcomes as those cited in ii–iv above. Induction of fermentation (v) does not occur in this case, but may be functional when the faster NADH replenishment comes from increasing glucose concentrations [71]

It was apparent that the following conditions are all able to slow down oxygen consumption and cell growth, and concomitantly, trigger carotenoid synthesis by *P. rhodozyma*: (i) nutritional cues such as copper deficiency; (ii) presence of respiratory inhibitors that interfere with electron transport at or downstream of complex III; and (iii) gene mutations impairing respiratory activity. This suggested that a common mechanism might account for triggering the synthesis of astaxanthin under these dissimilar conditions. Notably, the conditions i–iii, although not sharing a common target, nevertheless all affect targets on the oxidative branch of the main respiratory chain. Moreover, it is evident that anyone of these conditions could lead successively to decreased oxygen consumption, increasing pO_2_, and likely to a redox imbalance favoring a higher degree of reduction of the electron carriers of mitochondrial respiratory chain. Since the conditions cited also encourage the synthesis of carotenoids, each one must encourage the generation of O_2_^·−^/H_2_O_2_ (i.e., lead to oxidative stress) as well. Therefore, the mechanism that triggers astaxanthin synthesis under conditions ii and iii may be analogous to the mechanism that triggers astaxanthin synthesis under copper deficiency (i). Such a mechanism would comprise the inhibition of oxygen consumption by an effector, leading to increasing pO_2_ together with a redox imbalance, and thereupon to greater O_2_^·−^/H_2_O_2_ generation and synthesis of astaxanthin. Figure 4 summarizes the analogous events that account for the onset of astaxanthin synthesis under the three divergent conditions cited above.

As argued previously, a similar succession of events most likely occur at the diauxic shift, as a consequence of a NADH surplus derived from ethanol reassimilation. A rapid NADH replenishment resulting from ethanol reassimilation could presumably disturb the balance of NADH/NAD^+^, slow the activity of Krebs cycle and respiratory activity [68], leading to lower oxygen consumption as evidenced by the relatively high pO_2_ levels (Fig. 1a, b). These events together apparently prompted the generation of O_2_^·−^/H_2_O_2_, as suggested by the accumulation of astaxanthin at this stage. Thus, the mechanism triggering astaxanthin synthesis by *P. rhodozyma* shortly after the diauxic shift bears resemblance to the one that triggers astaxanthin synthesis under conditions i–iii.

In summary, the succession of events leading to astaxanthin synthesis arise by either of two circumstances: excessive NADH provision; e.g., from a fast NADH replenishment while consuming ethanol (or by increasing glucose concentrations, see Kaspersky [71]), or through several conditions that impair electron flow through the main respiratory chain. Importantly, a number of lines of indirect evidence suggest that redox imbalances, regardless of whether they result from excessive replenishment or hampered oxidation of NADH, together with the pO_2_ level, dynamically encourage O_2_^·−^/H_2_O_2_ generation in vivo by *P. rhodozyma*. In the past 15 years the role of O_2_^·−^/H_2_O_2_ has been gradually shifting from molecules that cause cell damage, to signals that mediate adaptations of cellular functions and metabolism to changing environmental conditions [51, 98, 99]. The dynamic tuning of this redox signaling, depending on its intensity and duration, can be a driving force to modify, via oxidation, the redox state of numerous target intracellular proteins [100–102]. Accordingly, *P. rhodozyma* cells subtly adjust their metabolism to the availability of nutrients, their genetic makeup, or to presence of respiratory inhibitors, using a common mechanism that depends on redox imbalances as shared event.

### The mechanism signaling redox imbalance in *P. rhodozyma* may be applicable to other cell types

Notably, even non-carotenoid producing yeasts, to all appearances function similarly with respect to respiratory response. For example, respiratory deficient strains of *S. cerevisiae* showed a 4-fold increase of NAD(P)H fluorescence compared to native yeast strains [103]. *Candida albicans* and *Candida dubliniensis* exhibited increased levels of NADH fluorescence whereas ATP decreased under the presence of respiratory inhibitors such as cyanide and antimycin A [104]. In addition, impairment of the electron transport flow can lead to a more reduced redox state in other cell types as well [46, 47, 49, 105–108]. Therefore, a slow rate of NADH oxidation, largely determined by respiratory activity, may cause imbalances leading to dynamic changes of the redox state of the NADH/NAD^+^ couple in many cell types. Our work indicates that redox imbalances (particularly of the NADH/NAD^+^ and QH_2_/Q couples) could be modulated, in vivo, by either nutrient level, genetic makeup (or mutations), or the presence of respiratory inhibitors. Thus, the mechanism disclosed in this study for signaling copper limitation might be applicable to a much broader set of environmental cues (beyond nutritional), genetic conditions and cell types than those described herein.

## Conclusions

The means by which cells sense and respond to environmental cues or to mutations that disturb their metabolism persist as a fundamental question of biological research [109, 110]. This work shows that copper deficiency and ethanol reassimilation, among others, represent events that can impair both oxygen consumption and growth. Any of these events appears to lead to similar outcomes: slow rate of oxygen consumption, increasing pO_2_ together with an impaired NADH oxidation, enhanced ROS generation, and consequently astaxanthin synthesis and increased *aox* expression. This suggests that the mechanism for triggering *asy* and *aox* expression, as well as astaxanthin synthesis under copper deficiency, is only one instance of a mechanism that integrates varied nutritional cues, as well as the presence of respiratory inhibitors, and genetic traits for tuning the release of O_2_^·−^/H_2_O_2_ and to generate a dynamic redox signal. Moreover, an analog mechanism can explain the induction of alcoholic fermentation regardless of whether aerobic o anaerobic. Furthermore, it is also significant that the analogous mechanism signaling each of the above conditions does not appear to be limited to *P. rhodozyma*. Instead, it could be operative in other cell types, including those of higher eukaryotes, to subtly adjust metabolism to their genetic makeup and to diverse and ever changing environmental conditions. Respiratory activity, through its effects on the redox state of electron carriers (e.g., NADH/NAD^+^ and QH_2_/Q couples) and pO_2_, appears to act as the master regulator of cellular metabolism, operating through a mechanism similar to the one disclosed here for *P. rhodozyma* growing under conditions of copper limitation. The mechanistic framework developed from own data, and from others, thus provides a sound basis and motivation for further research.
